# Predictors of emotional distress a year or more after diagnosis of cancer: A systematic review of the literature

**DOI:** 10.1002/pon.4601

**Published:** 2018-01-10

**Authors:** Sharon A. Cook, Peter Salmon, Gemma Hayes, Angela Byrne, Peter L. Fisher

**Affiliations:** ^1^ Institute of Psychology, Health and Society University of Liverpool Liverpool UK; ^2^ The Royal Liverpool and Broadgreen University Hospitals NHS Trust Liverpool UK; ^3^ Nidaros DPS Østmarka University Hospital Trondheim Norway

**Keywords:** anxiety, cancer, depression, emotional distress, oncology, predictor, prospective, systematic review, trauma

## Abstract

**Objective:**

Why some people recover emotionally after diagnosis and treatment of cancer and others do not is poorly understood. To identify factors around the time of diagnosis that predict longer‐term distress is a necessary step in developing interventions to reduce patients' vulnerability. This review identified the demographic, clinical, social, and psychological factors available at or within 3 months of diagnosis that are reliable predictors of emotional distress at least 12 months later.

**Methods:**

A systematic search of literature for prospective studies addressing our research question and predicting a range of distress outcomes was conducted. Thirty‐nine papers (reporting 36 studies) were subjected to narrative synthesis of the evidence.

**Results:**

There was no consistent evidence that demographic, clinical, or social factors reliably predicted longer‐term distress. Of the psychological factors examined, only baseline distress (significant in 26 of 30 relevant papers; 24 of 28 studies) and neuroticism (significant in all 5 papers/studies that examined it) consistently predicted longer‐term distress. The heterogeneity of included studies, particularly in populations studied and methodology, precluded meta‐analytic techniques.

**Conclusions:**

This review supports current clinical guidance advising early assessment of distress as a marker of vulnerability to persistent problems. Additionally, neuroticism is also indicated as a useful marker of vulnerability. However, the review also highlights that more sophisticated research designs, capable of identifying the psychological processes that underlie the association between these marker variables and persistent distress, are needed before more effective early interventions can be developed.

## 1 BACKGROUND

Despite improving prognosis, cancer is still a life‐threatening disease and diagnosis can have a profound emotional impact. Around half of all newly examined patients report clinically significant levels of anxiety and/or depression.^1^, ^2^ For most, distress resolves without specialist help,^3^ with most of this spontaneous improvement occurring between 4 and 13 months after diagnosis.^4^ However, there are some patients for whom distress does not decline spontaneously or who become distressed at a later stage,^4^, ^5^ with many long‐term survivors remaining at risk of clinically significant distress. Around a third of patients in treatment or long‐term follow‐up report levels of distress, including anxiety and/or depression, that warrant intervention.^6^ Annual prevalence of major depression or generalised anxiety disorder remains 22% in the fourth year after breast cancer diagnosis,^7^ while lifetime prevalence of cancer‐related post‐traumatic stress disorder (PTSD) is 10% to 12% for breast cancer and 20% for other cancers.^8^ Furthermore, a US population–based survey^9^ reported 6% prevalence of psychiatric disorders among cancer survivors—double that in the noncancer comparison group—even after controlling for socio‐demographic and clinical factors.

It is therefore unsuprising that psychological needs figureprominently among cancer survivors' concerns.^10^, ^11^ Unmet psychological needs compromise quality of life of patients and their families. In addition, they increase health care costs because distressed patients make more demands on both primary and secondary care resources.^12^, ^13^

Why some people recover emotionally after diagnosis of cancer and others do not is not well understood, even though the last 10 to 15 years has seen prolific research on psychological morbidity in cancer. Much of this research has focussed on quantifying prevalence and improving detection of emotional distress rather than identifying causal predictors.^14^ A smaller body of research has identified factors that are associated with persistent distress and that might therefore be implicated causally in maintaining it. Cross‐sectional studies of this kind, identifying clinical, socio‐demographic, and psychological correlates of distress, are, however, of limited value in identifying potential causal factors. For this, prospective research is more informative. We are aware of no existing synthesis of prospective research into predictors of persistent distress following cancer diagnosis. Therefore, the aim of the present study was to review prospective research that sought to identify variables available at the time of, or measured within 3 months of diagnosis that predict longer‐term distress (defined as at least 12 mo later).

## 2 METHOD

Methodology broadly followed the PRISMA statement^15^ for conducting and reporting systematic reviews.

### 2.1 Search strategy and selection criteria

The EBSCO electronic database, which encompasses 5 medical, nursing, and psychology databases (Medline full text, Psychinfo, PsychARTICLES, CINAHL plus, and AHMED), was systematically searched from inception to July 2017. We combined the term *cancer* with terms relating to emotional distress and those commonly used to denote prospective studies (see Table S1 for search strategy). In addition to generic terms used to denote emotional distress (ie, anxiety and depression), we included terms commonly used to describe persisting distress in response to a traumatic event such as cancer diagnosis (ie, post‐traumatic stress and adjustment disorder) and one arising specifically in the context of cancer (fear of cancer recurrence). Only English language papers were included. References of all papers retrieved were searched to ensure that relevant studies had not been missed.

### 2.2 Inclusion and exclusion criteria

Included studies (1) used a prospective cohort design, (2) were published quantitative studies examining predictors (≤3 mo after diagnosis) of subsequent emotional distress (≥12 mo later), (3) presented results for adult cancer populations with primary nonmetastatic cancer separate from any other chronic conditions, and (4) used published and validated outcome measures for emotional distress.

### 2.3 Data extraction

Study screening was shared by 3 authors (S.C., A.B., and G.H.) who worked independently and consulted where necessary to resolve ambiguous decisions. Study titles and, where necessary, abstracts were screened according to the inclusion criteria. Full text of potentially relevant articles was retrieved and screened. Data from eligible studies were extracted using a standardised protocol (Appendix A) and tabulated. Extracted data included general study details (author, date, and country), participants' details (age, gender, and cancer diagnosis), study design and methodology (sample size and attrition, outcome and predictor variables, timing of baseline and follow‐up assessments, and analysis method), and a summary of the reported findings (relevant beta coefficients or odds ratios and/or percentage variance explained). Data were extracted from each included article independently by one of two authors (S.C. or G.H.) with a reliability check for which 10% (selected at random) of papers were also subject to data extraction by the other author. There were no disagreements.

### 2.4 Data synthesis

Meta‐analytic review was considered inappropriate because the predictor variables and indices of distress varied greatly across the studies. Therefore a narrative synthesis is provided.

Findings for each distress outcome (anxiety and depression case, anxiety and depression symptoms, trauma symptoms, emotional distress symptoms) are reported within three broad categories of predictor variable: demographic and clinical; social; and psychological. In making inferences about the reliability of prediction by any one variable, we attended particularly to the number of studies in which that variable had been tested and the proportion in which it was significant.

## 3 RESULTS

**Figure 1:**
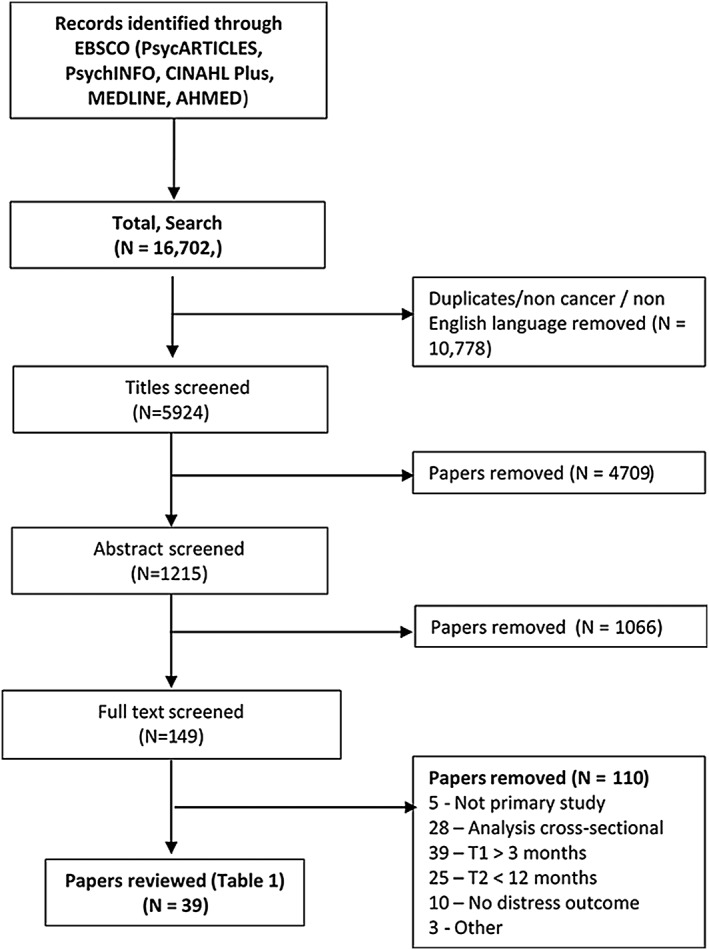
Flow diagram of study selection procedure

The search yielded 16 702 papers. After removing duplicates, 4709 papers were then removed by title and a further 1066 by abstract. The remaining 149 full‐text papers were retrieved and read with a final 110 excluded as a result (see Figure 1).

Thirty‐nine papers reporting 36 primary studies were included. Table 1 indicates study sample characteristics; Table S2 summarises study design and findings, grouped by type of distress. Table S3 provides a glossary of measures used to assess distress.

**Table 1:** Sample characteristics of 39 included papers (36 studies)

Paper	Diagnosis	% Female	T1 Sample N	T2 Sample N	Attrition (%)	Age Mean (SD)	Age Median (Range)	Country
Dean^16^	BC	100	122	111	9	48.7	(20‐60)	UK
Ramirez et al^17^	BC	100	102	91	11	‐	56 (24‐69	UK
Carver et al^18^	BC	100	66	61	8	52.9 (11.2)	(28‐76)	USA
Tjemsland et al^19^	BC	100	106	91	14	‐	50 (33‐70)	Eur
Hammerlid et al^20^	HN	28	357	215	40	63	(18‐88)	Eur
Bleiker et al, 2000^21^	BC	100	244	200	18	51.9 (10.5)	(29‐75)	Eur
De Leeuw et al^22^, ^a^	HN	21	204	155	24	59 (10.8)	‐	Eur
De Graeff et al^23^, ^a^	HN	20	204	153	25		(29‐76)	Eur
De Leeuw^24^, ^a^	HN	22	204	171/139/123	16‐40	59 (10.6)	‐	Eur
Ranchor et al^25^	Mix	42	167	99	41	73.4 (7.46)	‐	Eur
Stanton et al^26^	BC	100	80	70	12.5	52.6 (11.94)	30‐80	USA
Mehta et al^27^	PC	0	519	259	50	64.8 (4.8)	‐	USA
Shroevers, Ranchor, & Sanderman^b^, ^28^	Mix	73	475	403	15	58(14.3)	‐	Eur
Shroevers, Ranchor, & Sanderman^b^, ^29^	Mix	73	475	403	15	58(14.3)	‐	Eur
Uchitomi et al^30^	LC	40	262	212	19	62.1 (10.8)	63.5 (22‐83)	Japan
Schou et al^31^	BC	100	195	165	15	56 (10.3)	21‐78	Eur
Aarstad et al^32^	HN	0	27	27	0	59.9 (1.3)	‐	Eur
Millar et al^33^	BC	100	371	279	25	59.4 (10.9)	29‐98	Eur
Steginga et al^34^	PC	0	111	104	6	61.54 (8.13)	‐	Eur
Gustavsson‐Lilius et al^35^	Mix	68	349	123	65	58 (8.6)	34‐76	Eur
Lebel et al^36^	BC	100	146	86	41	61‐7 (10.8)	37‐88	Can
Barez et al^37^	BC	100	129	101	22	48.03 (8.4)	25‐65	Eur
Den Oudsten et al^38^	BC	100	223	144	35	58.7 (9.4)	‐	Eur
Ristvedt & Trinkaus^39^	RC	44	123	80	35	67.5 (12)	29‐88	USA
Couper et al^40^	PC	0	211	175	17	66.2 (8.3)	43‐92	Aus
Scharloo et al^41^	HN	24	177	95	46	59.6 (10.8)	36‐84	Eur
Elklit & Blum^42^	BC	100	81	64	25	56.3(9.1)	41‐89	Eur
Lee et al^43^	BC	100	299	206	31	‐	20‐79	Korea
O'Connor et al^44^	BC	100	3318	2912	7	‐	55.7 (26‐70)	Eur
Carlson et al^45^	Mix	43	877	505	42.5	62.3(14.1)	‐	Can
Lockefeer & de Vries^46^	BC	100	227	163	28	58.9 (9.3)	‐	Eur
Neilson et al^47^	HN	16	101	37	63	63	37‐85	Aus
Adachi et al^48^	HN	22	116	78	33	61.2(11.4)	20‐85	Japan
Hou & Lam^49^	RC	38	234	180	33	64.4(10.6)	67 (29‐82)	HK
Kohler et al^50^	PC	0	390	329	16	65.3 (6.4)	‐	Eur
Cook et al^51^	BC/PC	65	229	206	10	61,6 (9.0)	39‐58	UK
Stafford et al^52^	BC/GC	100	264	105	60	53.1 (13.0)		Aus
Pérez et al^53^	BC	100	126	102	19	50.5 (8.7)	27‐68	Eur
Saboonchi et al^54^	BC	100	750	750	0	51.3 (8.1)	52 (24‐63)	Eur

NB: BC, breast cancer; Mix, heterogeneous cancer diagnoses; HN, head and neck cancer; PC, prostate cancer; LC, lung cancer; RC, rectal cancer; GC, gynaecological cancer; UK, United Kingdom; Eur, Europe; USA, United States of America; Aus, Australia; Can, Canada; HK, Hong Kong.

a Three papers report one study.

b Two papers report one study.

Most studies were conducted in Europe (26 papers from 23 studies), 6 in North America (6 papers from 6 studies), and the remaining 7 (7 papers) in Australia, Japan, Hong Kong, and Korea. They predominantly reported breast cancer patients (19 papers from 19 studies), although head and neck, (8 papers from 6 studies), prostate (5 papers from 5 studies), rectal (2 papers from 2 studies), lung (1 paper from 1 study), gynaecologic (1 paper from 1 study), and heterogeneous cancer populations (5 papers from 4 studies) were also included. Mean sample ages ranged from 39 to 73 years. Reflecting the diagnostic groups studied, 18 of 39 papers (18/36 studies) reported samples that were entirely female.

Of the 36 primary studies, 2 included a premorbid baseline. For the others, baseline assessments were either just before participants received diagnosis (n = 2), immediately after diagnosis (n = 7), before primary treatment started (n = 15), or after primary treatment finished (n = 9).

The predominant indicator of distress was depression (22 papers from 20 studies). Nine of these papers (from 9 studies) also assessed anxiety. No paper reported anxiety alone. Six of these 22 papers (6 studies) tested prediction of anxiety and/or depression “caseness” or change in “caseness” at follow‐up; the remaining 16 (14 studies) predicted severity, or change in severity, of symptoms. Emotional distress or functioning was predicted in 13 papers (12 studies), fear of recurrence in 2 papers (2 studies), and trauma symptoms in 8 papers (8 studies).

Twenty‐seven papers (24 studies) assessed point prevalence of the outcome 12 to 18 months after baseline, 7 papers (7 studies) at 2 years, and 2 papers (2 studies) at 5 or more years after diagnosis. In addition, 4 papers (4 studies) assessed predictors of change in depression over the follow‐up period.

Most of the papers included multivariate analyses of predictors across more than one category (ie, demographic and clinical, social, and psychological). Some studies reported sequential analyses to reduce an inital set of potential predictors; in these circumstances, only data from the final analyses were included in this review. Most papers reported the results of logistic or multiple regression analyses using *P* < .05 to indicate a significant association, although there was considerable variation in method of entry and ordering of included predictors.

### 3.1 Socio‐demographic and clinical predictors

#### 3.1.1 Age and gender

Age was a significant predictor in only 4 of the 27 papers (25 studies) that assessed age effects. Younger age predicted trauma symptoms^19^ and emotional distress^26^ 12 months after breast cancer surgery; it also predicted anxiety and depression, but not trauma symptoms, 12 months after pretreatment assessment for breast and prostate cancer,^51^ and anxiety 18 months after diagnosis of head and neck cancer.^47^

In the 16 papers (13 studies) reporting mixed‐gender samples, 13 tested the effect of gender but only 2 (1 study) found it a significant predictor. Female gender predicted emotional distress but not depression 12 months after the start of treatment for head and neck cancer.^23^ However, in a second paper reporting the same study, female gender did predict depression 2 and 3 years post‐treatment (emotional distress was not reported at these times).^24^

#### 3.1.2 Socioeconomic status

Only 3 other demographic variables (education, income, and social class) were significant predictors in any study. Twelve papers (12 studies) tested the effect of education, but 9 papers found no effect. In one clinically heterogeneous cohort, patients with more education (not clearly defined) became less depressed from 3 to 15 months following diagnosis.^28^ However, this study only tested clinical and demographic factors, so it is unclear whether educational level would remain significant if social or psychological factors were also included. In lung cancer patients,^30^ lower educational level predicted depression, but not emotional distress, 12 months after treatment. In breast cancer patients,^54^ low educational level independently predicted anxiety, but not depression, 2 years after surgery.

Social class was tested in two papers (two studies) but an effect was found in just one, in which lower class (not clearly defined) predicted anxiety or depression “caseness” 12 months after mastectomy for breast cancer.^16^ Personal income was tested in just one study,^44^ in which lower income predicted severe trauma symptoms 15 months after surgery for breast cancer. This study again tested demographic variables separately from clinical and other factors.

#### 3.1.3 Clinical, treatment, and tumour characteristics

Eight of 10 papers (8 of 10 studies) testing treatment type in breast cancer found no effect either of type of surgery or of type of adjuvant therapy on distress outcomes. Of the two papers that did report effects, one^38^ found that undergoing breast‐conserving surgery rather than mastectomy or no surgery predicted depression 12 months after diagnosis. The other^19^ found no effect of surgery type, but having radiotherapy predicted fewer trauma symptoms at 12 months. In other cancer populations, just 2 of 10 papers (2 of 7 studies) that explored treatment as a predictor of distress outcomes reported an effect. De Graeff et al^23^ stated that combination therapy vs single treatment modality predicted emotional distress and depression 12 months post‐treatment for head and neck cancer. However, treatment was just one element of a composite variable aggregating tumour site, stage, and treatment and, in two further papers reporting the same study,^22^, ^24^ treatment type did not predict depression when considered independently. A more recent study of a clinically heterogeneous population^45^ reported that having surgical treatment predicted worse emotional distress over the 12 months since diagnosis, while having radiotherapy predicted worse depression and anxiety.

Of the 12 papers (10 studies) assessing disease‐related characteristics (stage, size, site, and nodal status), 8 (8 studies) found that these did not predict distress. One paper in a clinically heterogenous sample^28^ did report that more advanced disease predicted more depression 15 months after diagnosis. In a study of breast cancer patients,^44^ greater nodal involvement predicted severe symptoms of psychological trauma 15 months after surgery. Another study^22^, ^23^ found that cancer stage predicted depression 12 months after treatment for head and neck cancer when entered into the regression model before other pretreatment variables.^22^ However, this effect disappeared after controlling for treatment type and recurrence.^24^

#### 3.1.4 Physical health

Half of the 12 papers (5 of 10 studies) that tested physical health status as a predictor of emotional distress found an effect. Three studies in breast cancer found that worse prediagnosis physical health predicted trauma symptoms^19^ and worse prediagnosis fatigue predicted depression.^38^, ^46^ Another study (2 papers) found that poorer pretreatment physical functioning predicted worse depression and emotional distress 12 months after treatment for head and neck cancer.^22^, ^23^ However, a third paper reporting this study found no effect after controlling for treatment type and recurrence.^24^ Finally, postoperative sleep and health complaints predicted more trauma symptoms (ie, intrusive thoughts) 18 months after surgery for breast cancer.^21^

In summary, with the exception of baseline physical health (which had roughly equal numbers of significant and null findings), there is scarce evidence that baseline demographic or clinical factors predict longer‐term distress after cancer diagnosis.

### 3.2 Social predictors

Twenty‐one of 39 papers (19 studies) explored social factors as potential predictors of distress.

#### 3.2.1 Relationship status and social network

Ten of 11 papers (11 studies) exploring the effect of relationship status or living alone found no effect on long term distress. Just one^16^ reported that, 12 months after mastectomy, married women were more likely than single women to be classified as anxiety or depression cases.

One paper in head and neck cancer^24^ examined the influence of social networks and reported that a smaller formal social network (eg, doctor, nurse, and psychologist) predicted patients who became depressed 12 months after treatment, and a smaller informal social network (ie, partner, family, and friends) predicted those who became depressed at 3 years.

#### 3.2.2 Perceived social support

Four of 10 papers (3 of 9 studies) examining social support found it predicted distress. In one head and neck cancer study, less available support predicted depression 1 year after treatment after controlling for baseline depression^22^ while less emotional support predicted depression 1 to 3 years after treatment.^22^, ^24^ In a mixed cancer diagnosis cohort, after controlling for depression, a lack of “problem‐focussed” support and more negative interactions with others 3 months after diagnosis (but not emotional support) predicted depression 12 months later.^29^ In one study, in a mixed cancer diagnosis cohort, more supportive social interactions before diagnosis predicted greater emotional distress 12 months after diagnosis.^25^

#### 3.2.3 Negative life events

Just 3 papers (3 studies) examined whether distress was predicted by negative life events before cancer^19^, ^21^ or by previous serious illness,^31^ and all found no effect.

In summary, there is little evidence to suggest baseline social factors are useful predictors of longer‐term distress.

### 3.3 Psychological predictors

#### 3.3.1 Emotional distress

Most of the reviewed papers (30 out of 39) examined whether baseline measures of distress predicted distress at follow‐up. In most cases, the same measure of distress was used on both occasions and was the largest or only significant predictor. Just 4 papers (4 studies) reported that baseline distress did not predict follow‐up distress. In 3 of these, depression prediagnosis^46^ or at diagnosis^31^, ^32^ did not predict depression at follow‐up (24, 12, and 72 mo later, respectively). The remaining negative finding arose from a study of emotional distress after breast cancer with a follow‐up of 6 years.^36^

Four papers (4 studies)^17^, ^19^, ^44^, ^52^ used different measures of distress at baseline and follow‐up. Three found positive effects. In one, preoperative emotional distress predicted anxiety or depression “caseness” 12 months after surgery for breast cancer.^17^ Another reported that having received treatment for anxiety or depression before diagnosis of breast or gynaecological cancer predicted depression 12 months later but not anxiety. However, this variable predicted neither outcome at 18‐ nor 24‐month follow‐up.^52^ In the same study, being in treatment for anxiety or depression at the time of diagnosis predicted anxiety 12 and 18 months later and depression at 18 months, but neither outcome at 2 years.^52^ The third paper to find an effect of distress assessed at baseline reported that premorbid psychiatric history (diagnoses unspecified) predicted severe trauma 15 months after surgery for breast cancer.^44^ The final paper,^19^ found that preoperative diagnosis of PTSD did not predict trauma symptoms 12 to 16 months after surgery for breast cancer.

#### 3.3.2 Self‐esteem

Only one paper reported data on self‐esteem and found no effect of prediagnosis self‐esteem on 12‐month depression in breast cancer patients.^38^

##### Coping

Twelve papers (11 studies) examined coping, using a variety of measures to assess cognitive and behavioural strategies.

Five papers (5 studies) across breast,^16^, ^33^, ^42^ prostate,^34^ and head and neck^48^ cancer found no effect of coping. One paper, in head and neck cancer, claimed small benefits of pretreatment coping through religion 12 months later and small benefits of trying to ameliorate pretreatment emotional distress 3 years later.^24^ However, another paper analysing the same data found no effect of any aspect of coping at 12 months.^22^

Coping did predict emotional distress, even after controlling for baseline distress, in the remaining 5 studies, although findings were diverse. Pretreatment “fatalism” predicted depression, but not anxiety, 12 months later in prostate cancer,^40^ whereas fatalism at diagnosis predicted anxiety caseness but not depression caseness 12 months later in breast cancer.^31^ “Helpless/hopeless” coping predicted depression caseness 12 months after breast cancer diagnosis.^31^ One study reported that specific coping styles predicted cancer patients' trauma symptom trajectories in the first year after diagnosis.^53^ This study identified 4 different trajectories of trauma symptoms on the basis of latent growth mixture modelling: resilient (low levels of symptoms at each measurement), delayed‐recovered (initial low levels of symptoms that increased and then decreased), mild (initial moderate symptoms slowly decreasing over time), and chronic (persistent high levels of trauma symptoms). Coping by “anxious preoccupation” predicted “mild,” “chronic,” and “delayed‐recovered” symptom trajectories. Cognitive avoidance predicted a “mild” trajectory. Preoperative “acceptance” predicted less emotional distress 12 months after surgery for breast cancer.^26^ Finally, “active problem solving” and “positive reframing” 3 months after breast cancer diagnosis predicted greater emotional distress 6 years later.^36^

#### 3.3.3 Personality

Thirteen papers (13 studies) examined personality traits, measured around the time of diagnosis. Five of these (5 studies) assessed neuroticism, finding that it predicted distress. After controlling for baseline distress, it predicted 12‐month emotional distress^25^ in a group with mixed cancer diagnoses, and 12‐month emotional distress,^33^ depression,^38^, ^52^ anxiety,^52^ and trauma symptoms^19^ in breast cancer. In breast and gynaecological cancer, it also predicted depression at 18 months, and both anxiety and depression 24 months after surgery.^52^ Finally, in newly diagnosed head and neck cancer patients, controlling for neuroticism reduced to nonsignificant the correlation between baseline depression and depression at 6 years (the independent contribution of neuroticism to predicting depression was not reported).^32^

Optimism‐pessimism was assessed in 5 papers (5 studies). In breast cancer,^31^ pessimism at diagnosis predicted anxiety and depression caseness 12 months later after controlling for baseline distress, while postoperative optimism predicted lower levels of anxiety and depression 2 years after surgery.^54^ However, 3 other papers in prostate^34^ and breast cancer^21^, ^36^ reported no effect after controlling for baseline distress.

“Type C” personality was assessed in one paper that explored predictors of 4 different trauma symptom trajectories in the first year after diagnosis of breast 53 (see explanation above). It predicted a “mild” trajectory.

When it was the only personality variable entered in regression models, trait anxiety predicted emotional dysfunction 2 to 5 years after surgery for rectal cancer,^39^ and depression 2 years after diagnosis of breast cancer.^46^ However, it did not predict depression 12 months after diagnosis if other personality measures were controlled for (neuroticism and agreeableness).^38^

#### 3.3.4 Perceived control

Despite the salience of perceived control in psycho‐oncology, only 6 papers (5 studies) examined this variable, with just 3 finding positive effects. In breast cancer, preoperative “personal control” did not predict emotional distress 12 months later,^33^ although greater postoperative perceived control (a latent variable inferred from scores on “fighting spirit” and “self‐efficacy”) did predict improvement in emotional distress over the subsequent year.^37^ In a sample of breast and prostate cancer patients, pretreatment personal control predicted anxiety 12 months later but not depression or trauma symptoms.^51^ In another study with mixed cancer diagnoses,^35^ “sense of coherence” (a similar construct to perceived control) at diagnosis predicted lower anxiety and depression 14 months later. In head and neck cancer patients,^22^, ^24^ cancer locus of control (a sense of personal control over the cause and course of cancer) had no relationship with depression 1 to 3 years after treatment.

#### 3.3.5 Illness/treatment perceptions

Three of 5 papers (5 studies) that explored whether patients' appraisal of their illness predicted longer‐term distress used a standardised measure of illness perceptions while the remaining two used single‐item measures. Only two found any effects. In one^33^ of 4 illness perception factors assessed only postoperative “illness identity” (perceived symptom burden) predicted emotional distress 12 months later among breast cancer patients. In the second, after controlling for baseline distress, just 1 of 8 illness perception factors (“personal control”) predicted anxiety, but not depression or trauma symptoms, after treatment for breast or prostate cancer.^51^

#### 3.3.6 Metacognitive beliefs

One paper^51^ examined whether metacognitive beliefs (ie, positive and negative beliefs about cognition) predicted distress 12 months after treatment for breast or prostate cancer. After controlling for pretreatment distress and illness perceptions, 1 of 5 metacognitive subscales (“cognitive confidence”) predicted anxiety and depression, but not trauma symptoms.

In summary, of the psychological factors examined, only baseline distress and neuroticism consistently predicted longer‐term emotional distress.

## 4 DISCUSSION

This study is the first comprehensive review of prospective predictors of longer‐term distress after cancer. The specific aim was to identify variables measured before, or within 3 months of, cancer diagnosis that predicted distress at least 12 months later. A systematic search of literature identified 39 papers reporting 36 studies that examined a wide range of clinical, demographic, social, and psychological variables. Only 2 variables consistently predicted longer‐term distress after cancer: baseline level of distress, supported in 26 of 30 papers (24 of 28 studies) that examined this variable, and neuroticism (supported in 5 of 5 papers/studies). All the other putative predictor variables have either been examined in fewer than 4 papers/studies and/or were significant in ≤50% of the papers that tested them.

Although socio‐demographic risk factors for distress after cancer are often suggested to be similar to those in the general population,^7^ the predominance of negative findings in this review suggests that they are not important predictors, at least when other variables are included in analyses. Similarly, despite the popular belief that clinical factors (ie, treatment type and tumour characteristics) are likely to influence levels of distress, we found no clear evidence to support this view in the longer‐term. The only clinical variable for which there was modest evidence was premorbid physical health. However, even in this case, the findings were inconsistent, with as many papers finding no effect (ie, 6 of 12 papers or 5 of 10 studies) as those reporting significant prediction. This lack of association between clinical factors and distress is consistent with ideas from health psychology and psycho‐oncology research that emotional distress is more closely linked to individuals' appraisal of their clinical condition and context rather than to the clinical factors per se.^33^, ^41^, ^55^, ^56^, ^57^ However, this review also found no consistent evidence that measures of illness appraisal predicted longer‐term distress.

Despite the long‐standing view that social factors, in particular social support, protect against distress,^7^, ^58^, ^59^ we found no evidence that social support variables reliably predicted longer‐term distress. It may be that null findings reflect the difficulty of disentangling the effects of social support from other factors around the time of diagnosis, particularly baseline distress, which was controlled in most studies and with which social support is likely to be highly correlated. Alternatively, the inconsistent results reported across studies in relation to perceived social support may be due in part to the different ways that this variable was operationalised. The fact that one paper^25^ reported a direction of effect contrary to expectation (more baseline social interaction predicted greater emotional distress 12 mo later) suggests a need to revisit the construct of “social support” in future research and recognise it as complex and multidimensional.

Of the psychological variables studied, just baseline distress and neuroticism consistently predicted longer‐term distress. Studies of optimism/pessimism, perceived control, and self‐esteem provided no clear evidence for the reliability of these variables as predictors. Reports of significant prediction by measures of coping, illness perceptions, and metacognition should be interpreted with caution because significant findings arose for isolated subscales from larger questionnaires and might therefore be type 1 errors. In addition, the considerable variability in how coping and illness perceptions, in particular, were measured further compounds the difficulty of discerning any pattern in the findings reported across studies.

### 4.1 Strengths and limitations

This study is the first to use a rigorous and systematic approach to review research on prospective predictors of longer‐term distress after diagnosis and treatment of cancer. Narrative synthesis allowed us to include studies predicting a broad range of indicators of distress. However, the heterogeneity of included studies, particularly in the populations studied, the methods used to measure predictor and response variables, and procedures of data analysis, limits the conclusions that can be drawn.

Despite a comprehensive database search strategy, it is possible that some relevant research was missed. However, the main findings of the review are likely to be robust to missing studies; that is, given the general variability in methods and findings in this field, robust conclusions have to be drawn from findings across several studies rather than be based on isolated studies. Indeed, some variables (ie, self‐esteem and metacognitive beliefs) have been examined in only one paper; these variables therefore need further research to test and expand the preliminary findings that have been reported before any conclusions can be reached.

### 4.2 Clinical and research implications

This review has several important clinical and research implications. The most compelling finding that distress within 3 months of diagnosis predicts longer‐term distress (at least when the same measure is used) shows that, for many patients, distress is a persistent problem. This finding supports current guidance for assessing distress around diagnosis as a marker of those vulnerable to longer‐term distress.^10^, ^11^ However, it cannot be assumed that it is always necessary or even appropriate to treat distress detected so soon after diagnosis.^14^ Indeed, recent reports have suggested that patients do not necessarily want such early intervention,^60^ and PTSD literature has suggested that early intervention can do more harm than good.^61^, ^62^ Furthermore, although baseline distress may be a useful marker of future vulnerability, it remains unknown how or why distress is maintained for some patients and not others. In standard multivariate regression analyses of the kind used in much of the literature reviewed here, baseline distress inevitably dominates in predicting future distress and so might mask the predictive effect of other measured baseline variables that could be important in causing distress to persist.^51^ In the present review, one study used advanced statistical methodology to separate the enduring component of distress from the change in distress over time. Using latent growth curve analyses, this study reported 3 significant predictors of change in distress: baseline distress (intercept), baseline perceived control, and change in perceived control (slope). It found that the rate of change in perceived control (ie, slope) was the strongest predictor.^37^ Use of such advanced statistical approaches will provide greater opportunity for identifying the putative causal variables that underlie persistent distress.

Neuroticism emerged from the review as the second consistent indicator of vulnerability. Furthermore, the specific finding by Aarstad et al^32^ that neuroticism reduced to nonsignificant the effect of depression at diagnosis on depression at 6 years implies that enduring characteristics of the individual, rather than the more transient emotional responses, appraisals, or coping strategies that arise around the time of diagnosis, explains why distress persists. However, until the psychological mechanisms underlying the association between neuroticism and persistent distress are clearer, this finding is of limited help in guiding intervention.

This review highlights important gaps in the literature. In particular, several of the predictor variables examined (ie, social class, income, social network, negative life events, self‐esteem, Type C personality, trait anxiety, and metacognitive beliefs) were tested in fewer than 4 papers. Consequently, there is insufficient evidence to determine whether these are relevant predictors; these understudied variables should be tested in future research.

From our review, it is clear that, to understand longer‐term distress after cancer diagnosis, research is required that both acknowledges and controls for the persistence of distress while also seeking to identify the variables that have a causal role in maintaining distress. Such research requires a strong theory‐driven approach. This review suggests that there is currently only limited evidence to support the role of factors such as appraisal and coping, which are key components of traditional cognitive models of adjustment in cancer. Emerging models in mental health, such as relational frame theory^63^ or the metacognitive model,^64^, ^65^ provide an alternative direction for research. This review found only one study that drew on such approaches, reporting that metacognitive beliefs predicted longer‐term distress.^51^ As an isolated finding it is premature to draw conclusions from it in the context of this review. Nevertheless, further exploration of metacognitive and other theoretically derived potential causal variables is now needed.

## 5 CONCLUSION

This review found that distress and neuroticism, measured around the time of cancer diagnosis, are the only consistent indicators of vulnerability to long‐term emotional distress that have, until now, been identified. However, to understand the causes of this vulnerability and to develop interventions to reduce vulnerability, research needs to identify the psychological factors that maintain distress, and not just those that predict it. To achieve this, future research, based on testable theory, will need to adopt more sophisticated longitudinal designs and statistical methodology so that it can disentangle the persistence of distress from its causation.

## CONFLICT OF INTEREST

The authors have no competing interests to report.

## Supporting information

Table S1: EBSCO database search strategy

Table S2 Summary of study design and significant findings from included papers (grouped by outcome (DV))

Table S3: Glossary of distress measures (DVs) used in included papers

## APPENDIX A.

### A..1

Data Extraction Sheet

Form for the extraction of information from primary studies:

*Predictors of persistent distress after cancer*

Study

Author(s):

Source:

Date:       Vol:  Part:  Pages:

Research Question:

Study Sample

Target population:

    Country:           Ethnicity:

    Gender:           Age:

    Employment/Educ Status:

    Diagnosis:          Treatment.

Data collection

Setting:   Home/Clinic/other (*specify*)

Method:  Interview / Questionnaires

Outcome Variables Assessed (*Measures used*):

    Anxiety         Y/N

    Depression       Y/N

    PTSD symptoms     Y/N

    QoL (mental health scale) Y/N

    Other (*specify*)      Y/N

Adequacy of outcome measures employed:

Predictor Variables Assessed (list with measures)

    Medical       Y/N

    Social/environmental Y/N

    Psychological    Y/N

Design

    Prospective cohort

    Cross‐sectional Cohort (historical predictors?)

Timing:   Baseline assessment:

   Follow‐up assessment(s):

Sampling Method:

   Entry and exclusion criteria:

Is sample representative of study population Y/N

Sample size:

   Baseline response rate:

   Follow‐up response rate:

   Attrition:

**Results**

Were the basic data adequately described? Y/N.

Quantitative:

   Analysis used:

   Statistical findings reported (*list*)

   Statistical information omitted.

**Discussion**

Authors conclusion:

Reviewers comments

Include / Exclude

Reason:
