# Supplementary material for: Predictors of emotional distress a year or more after diagnosis of cancer: A systematic review of the literature
Source: Psychooncology. 2018 Jan 10;27(3):791–801. doi: 10.1002/pon.4601 (PMC5873392; doi:10.1002/pon.4601)
Supplement: Supplementary file 1 — Table S1: EBSCO database search strategy [file PON-27-791-s001.doc]

Table 1: EBSCO database search strategy

| **Connector** | **Search Term** | **Search field** |
| --- | --- | --- |
|  | cancer | All fields |
| **AND** | | |
|  | emotional distress OR psychological distress OR anxiety OR depress* OR posttraumatic stress OR PTSD OR psychological morbidity OR psych*, adjustment OR emotional adjustment OR mood OR adjustment disorder OR acute stress disorder OR fear of recurrence OR distress | All fields |
| **AND** | | |
|  | predict* OR risk factorsOR caus* OR vulnerability | All fields |
| **NOT** | | |
|  | adolescent cancer OR child* cancer OR paed*carers OR palliative | Abstract |
| **NOT** |  |  |
|  | genetic testing OR genetic screening | Abstract |
| **NOT** |  |  |
|  | palliative OR metastatic cancer ORadvanced cancer OR survival OR mortality | Title |
