# Supplementary material for: Predictors of emotional distress a year or more after diagnosis of cancer: A systematic review of the literature
Source: Psychooncology. 2018 Jan 10;27(3):791–801. doi: 10.1002/pon.4601 (PMC5873392; doi:10.1002/pon.4601)
Supplement: Supplementary file 2 — Table S2 Summary of study design and significant findings from included papers (grouped by outcome (DV)) [file PON-27-791-s002.doc]

**Table 3 Summary of study design and significant findings from included papers (grouped by outcome (DV))**

| **Article** | **Cancer Diagnosis** | **T1** | **T2 (N months later)** | **Dependant Variable (DV)** | **T1 DV controlled** | **Analysis** | **Medical /demographic** | **Social /Environmental** | **Psychological** | **Significant Findings (p<.05)** |
| --- | --- | --- | --- | --- | --- | --- | --- | --- | --- | --- |
| **DV - ANXIETY/DEPRESSION CASE** | | | | | | | | | | |
| Dean 1987 | Br | Pre-op | 12 | Anx or Dep case (PSE) | Y | Stepwise LR | Menopausal status, Trt, Social class, | Marstat, Confidant | Pre-op case (RDC/GHQ)  Coping style  Psy Trt  Attitude | Lower social class OR 4.57  Pre-op case OR 4.37  Perimenopausal OR 8.9  Marital status (married) OR 5.85  (results not clear) |
| Ramirez et al 1995 | Br | Pre-op | 12 | Anx or Dep case (PSE) | N | ROC |  |  | ED (HADS>10) | HADS >10 identified in 83% cases |
| Hammerlid et al 1999 | HN | Diag | 12 | Anx or Dep case (HADS) | Y | LR | KPS, Age, Gender, TSite, TStage | Living status | Baseline Anx or Dep case | Anx or Dep case at diagnosis **- *no data provided*** |
| Shroevers et al, 2003^*^ | Mix | Post-Trt (3 month post-diag) | 15 | Change in Dep case status over time (CES-D) | N | Repeated measures Anova | TSite, TStage, Trt, Age, Gender, Educ | Marstat |  | Greater reduction in Dep with lower stage disease (stage 1 vs stage 2 or higher) F [2,332], p<.05 and higher education F [3,332], p<.01 |
| Schou et al 2004 | Br | Diag | 12 | Dep case (HADS)  Anx case (HADS) | Y  Y | LR | Educ, Tgrade,Trt | Previous cancer/serious illness experience | Optimism /pessimism, +VE Trt expectation,  Anxi/Dep, Coping | **Dep Case:** low Opt OR = 0.83; Anxious preoccupation OR = 3.2  **Anx Case:** low Opt OR = 0.86; Anx OR = 2.71; fatalism OR = 3.16 |
| Uchitomi et al 2003 | Lc | Post-Trt  (1 mth) | 12 | MD Case(SCID)) | Y | LR (backward) | Age , Gender, Educ, Pre-op smoking, Pre-op TStage, Trt, dypsnea, Forced expiratory volume (FEV) | Marstat, | Pre-morbid/ pre & post Trt MD, post Trt ED | Post-Trt MDD OR = 2.1, Educ OR = 2.4 |
| **DV - ANXIETY/DEPRESSION** | | | | | | | | | | |
| De Leeuw et al 2000^**^ | HN | Pre-Trt | 12 | Dep (CES-D) | Y | Hierarchical MR (stepwise) | TStage Trt, Age, Gender, Symptoms, General health, Physical functioning | Received/available support, perceived social network | Coping, Locus of Control, Dep | TStage R^2^c =.0 4  Dep R^2^c =.20  Available supp R^2^c = .07  Social network R^2^c = .04  Gen Health R^2^c =.02 |
| De Graeff et al 2000^**^ | HN | Pre-Trt | 12 | Dep (CES-D) | N  Y | MR (stepwise) | Gender, Age , Group(site, stage, Trt), Karnovsky Performance Status (KPS), symptoms |  | Dep | Dep R^2^c = .21  KPS R^2^c = .03  Grp R^2^c =.02 |
| De Leuw et al 2001^**^ | HN | Pre-Trt | 12, 24,36 | Dep (CES-D) | Y | MR (stepwise) | Physical function , Symptoms, Trt, Recur, Tstage, Age, Gender | Received/available support, perceived social network openness to discussion | Dep, Coping, Locus of Control | Largest predictor Dep R^2^c =.18 @ 1yr  .31 @2yr  .31 @ 3 yrs  Coping R^2^c =.02 @ yr1 (Religious), .02 @ yr3 (Palliative coping)  Emotional support variables R^2^c = .14 @1 yr, .06 @ 2 yrs, .03 @ 3 yrs (incl. Social network & support)  Physical/dem og R^2^c = .01 @yr1, .02 @ yr 2, .10 @ yr 3 |
| Shroevers 2003^*^ | Mix | Post-Trt (3 month post-diag) | 15 | Dep (CES-D) | Y | MR (Stepwise) | Sociodemog (not stated) ,Group membership (patient vs. control) | Social support | Dep, Self-esteem | Dep β = 0.59  Problem focussed support β = 0.11  Negative interactions β = 0.09  Negative self-esteem (not shown ) |
| Aarstad et al 2005 | HN | Diag | 72+ | Dep (BDI) | Y | Partial Correlation |  |  | Anx; Dep ; Humour; | Dep r =0.39  Not sig after controlling for T1 neuroticism; Humour r = 0.42  r=0.64 after controlling for T1 neuroticism |
| Gustavsson et al 2007 | Mix | Diag | 14 | Anx (EMAS-State)  Dep (BDI) | Y | SEM - path analysis | Gender, Educ, TStage | Partner sense of coherence (SOC) | SOC (life as predictable/manageable/meaningful) Anxiety, Dep | **Anx DV:** Anx β =0.32, in direct effect of T1 SOC via T2 SOC β =-0.30, via T1 Anx β =-0.15  **Dep DV:** Dep β =0.36, in direct effect of T1 SOC via T2 SOC β =-0.30; via T1 Anx β =-0.22 |
| Den Oudsten et al 2009 | BC | Pre-diag | 12 | Dep (CES-D) | Y | MR (stepwise) | Age, Empstat, Educ, Surgery, Adj Trt, Tstage, Tsize, Fatigue, Pain & discomfort | Social support,  living with partner, living with children | Personality, Dep, trait Anx, Self-esteem, Body image, Cognitive function | Fatigue β =0.28, Neuroticism β =0.16, Surgery β =-17, Agreeableness β =-0.15 Dep 0.22 |
| Couper et al 2010 | PC | Pre-Trt | 12 | Dep  Anx  (BSI) | Y  Y | Hierarchical MR | HRQoL |  | Dep, Anx,, Coping | **Dep DV**: Dep β = 0.48, QoL-vitality β = 0.24, Fatalism β = 0.13  **Anx DV:** anx β = 0.62, QoL-vitality β = -0.19 |
| Lee et al 2011 | BC | Diag | 0-12 month | Deteriorated Dep (Zung-SDS) | N | Hierarchical LR (Cross-sectional) | Age, Co morbidity, smoking, Menopausal status, Deteriorated finances, Radiotherapy, Deteriorated role functioning | T1 predictors -  Deteriorated emotional support |  | No T1 Sig predictors. Deteriorated emotional support OR = 3.4, Deteriorated finances OR = 2.9, Deteriorated role functioning = 2.3 |
| Carlson et al 2013 | Mix | 1 month since diag | 12 | Improved Dep  (PSSCAN)  Improved Anx  (PSSCAN) | Y  Y | MR  (improved DV) | Age, Gender, Source of income , Educ, Ethnic/cultural background  TSite , Trt | psychosocial resources, marstat, livstat, | Anx, Dep | **Dep DV:** Dep β =-0.48, No radio β =0.08, psychosocial resources β =0.10,  **Anx DV:** Anx β =-0.42  Anx x married β =-0.11  Anx x no radio β =0.11  Anx x not gastro cancer β =-0.11 |
| Lockefeer & Defries 2013 | BC | Pre-diag | 24 | Dep (CES-D) | Y | Hierarchical MR | Age Educ, empstat, TSite, Chemo, Radio, HT, Fatigue, Sleep quality | partner, , children, | Dep, trait Anx | Trait anxiety β = 0.37, Fatigue β = 0.23 |
| Neilson et al 2013 | HN | Pre –Trt | 18 post Trt | Dep  Anx  (HADS) | N  N | Multi-level mixed effects linear regression | Time, Age, Gender, Chemo, Pain, Symptoms, Trt | livstat, |  | **Dep DV:** Symptoms β = -.24  **Anx DV:** Age β = 0.54, symptoms β = -0.09e |
| Adachi et al 2014 | HN | Pre-op | 12 | Dep (HADS) | Y | MR (stepwise) | Gender, Facial disfigurement | Social support | Dep, Coping, Trauma | Dep β =-0.59 |
| Cook et al, 2015 | BR/PR | Pre-trt | 12 | Dep  Anx  (HADS) | Y | Hierarchical MR | Age, Gender | - | Anx  Dep  Illness perceptions (inc. Personal control)  Metacognitive beliefs | Anx DV**:**  Age β = 0.18; anxiety β =0.46; Personal Control β = -0.11; Cognitive confidence β=0.13  Dep DV**:**  Age β = 0.14; depression β =0.41; Cognitive confidence β=0.20 |
| Stafford et al, 2015 | BC/GC | Pre-op | 12, 18, 24 | Dep (CES-D)  Anx (HADS) | N | Hierarchical MR | Age, Educ, TSite; Chemo, Radio | Living alone, | Psychiatric trt at diag (PTd)  Psychaitric treatment prior to diagnosis (PTh)  Neuroticism | Anx DV: neuroticism β = 0.31; PTd β = 0.24 @12 months ; PTd β = 0.31 @18 months ;  neuroticism β = 0.27; @24 months ;  Dep DV: neuroticism β = 0.21; PTh β = 0.32 @12 months ; neuroticism β = 0.31; PTd β = 0.26 @18 months ;  neuroticism β = 0.33; @24 months ; |
| Saboonchi et al, 2016 | BC | Post-op | 12,  24 | Dep  Anx  (HADS) | N | LGC | Age, Educ,  Adj trt | Marstat | Personality (optimism, Pessimism) | Dep DV (R^2^= .51): optimism intercept b = -1.01;  Anx DV (R^2^= .39): : optimism intercept b = -0.82, Educat b = .90 |
| **DV – TRAUMA SYMPTOMS** | | | | | | | | | | |
| Tjemsland et al 1998 | BC | Pre-op | 12-16 | Trauma (IES) | Y | MR | Age, Adj Trt, Health problem / Medications in last 10yrs, Recurrence | Work/social/family function, Lack of crisis support, Life events | Emotionality (EPI-N), Intrusion, PTSD casesness | Emotionality β = 0.45  Intrusion β =0.36  Medication β = 0.34  Adj Trt β = -0.17  Age β = -0.16 |
| Bleiker et al 2000 | BC | Post-op | 18 (21 after surgery) | Intrusion  Avoidance  (IES) | Y | MR (Backward) | Age, Surgery, Lymph nodes, Adj Trt, Sleep, Health complaints (SCL-90) | Life events, Perceived social supp (SEC) | Intrusion, Avoidance, Anx, Anger Dep, Personality (Optimism, Rationality, Anti-emotionality, emotional expression ) | **Intrusion DV:** T1 Intrusion β = 0.60  Health complaints β = 0.28  Sleep problems β = 0.25  **Avoidance DV,** T1 avoidance explained 47%no beta provided |
| Lebel et al, 2008** | BC | Post-Trt | 72 | Trauma (IES) | Y | Hierarchical MR (stepwise) | Age, Educ, 2nd cancer, Perceived health | Social support | Coping, Optimism, Fear of future, Stress appraisal , Emotional distress, Trauma | Trauma β =-0.52, 2^nd^ cancer β =-0.19 |
| Risvedt & Trinkaus 2009 | RC | Post-op | 24-60 | Trauma (IES) | N | LR | Gender, Age, Educ,TStage, Ostomy , Faecal incontinence |  | Trait anxiety | Faecal Incontinence OR 1.05 |
| Elkit & Blum 2011 | BC | Post op | 13 post diag | Trauma (HTQ) | N) | Hierarchical MR |  |  | Immature defence style, Emotional coping, Avoidance. Negative affectivity | Avoidance β =0.25, Negative affectivity β = 0.55 |
| O’Connor et al, 2011 | BC | Pre-morbid | 15 post-op | Trauma  (IES ≥35) | Y | LR | Step1: Age, Educat, Income, Net wealth, Ethnicity, Co-morbidity  Step 2: Nodal Involvement, Tstage, Tsize, Receptor status, Surgery, Chemo, Radio, Hormone Trt | Step 1: Marstat, Children, Urban | Step 1: Psychiatric History | Step 1: Income OR (95% CI) 0.64 (0.43-0.95), Net Wealth OR (95% CI) 0.50 ().33-0.75), Psychiatric History OR (95% CI) 1.78 (1.16-2.72)  Step 2: Nodal Status OR (95% ci) 1.81 (1.37-2.40) |
| Cook et al, 2015 | BC/PC | Pre-Trt | 12 | Trauma  (IES) | Y | Hierarchical MR | Age, Gender |  | trauma  Illness perceptions (inc. personal control)  Metacognitive beliefs | Trauma. β =0.44; |
| Pérez et al, 2016 | BC | Pre-op | 12 | Trauma (Symptom trajectory groups: Chronic, Mild, Delayed-recovered, Resilient)  (SASRQ) | Y | MLR (ref: Resilient) |  |  | Type C Personality, Coping | Mild: Anxious preoccupation β =0.30 OR (95% CI) 1.09-1.65; Cognitive Avoidance β =0.21 OR (95% CI) 1.01-1.51, Type C β =0.38 OR (95% CI) 1.07-1.98;  Chronic: Anxious preoccupation β =0.78, OR (95% CI) 1.27-3.89; |
| **DV - EMOTIONAL DISTRESS/FUNCTIONING** | | | | | | | | | | |
| Carver et al 1998 | BC | Pre-Trt | 12 | ED (Affects Balance Scale) | N | MR | Age |  | Body image , Appearance concern, Body integrity concern | None sig |
| De Graeff et al 2000^**^ | HN | Pre-Trt | 12 | EF (QLQ-C30-EF) | N  Y | MR (stepwise) | Gender, Age , Group(Tsite, Tstage, Trt), Karnovsky Performance Status (KPS), symptoms |  | Dep | Dep R^2^c= 17  KPS R^2^c=.0 4  Gender R^2^c = .02  Grp R^2^c = .01 |
|  |  |  |  |  |  |  |  |  |  |  |
| Ranchor et al 2002 | Mix | Pre-morbid | 12 | ED (GHQ-12) | Y | Hierarchical MR | Age, Gender, Educ | Social support | ED, Neuroticism , Self-Efficacy | ED β = 0.26, Neuroticism β = 0.22, Social support β = 0.38 |
| Stanton et al, 2002 | BC | Pre-op | 12 | ED (POMS)  FOR (Fear of Recurrence Scale) | Y  Y | Hierarchical MR | Age |  | Distress, Vigor, Coping, Hope, | **ED DV:** ED & age R^2^c = .30, Coping R^2^c = .14, Hope x coping R^2^c .21  **FOR DV** – FOR R^2^c = .46, Hope x coping R^2^c = .19 |
| Uchitomi et al 2003 | LC | Post-Trt  (1 mth) | 12 | ED (POMS) | Y | MR (backward) | Age , Gender, Educ, Pre-op smoking, Pre-op TStage, Trt, dypsnea, Forced expiratory volume (FEV) | Marstat, | Pre-morbid/ pre & post Trt MD, post Trt ED | Post-Trt distress β = 0.47, Pre-Trt MDD β = 0.18 |
|  |  |  |  |  |  |  |  |  |  |  |
| Millar et al 2005 | BC | Post-op | 12 | ED (GHQ-28) | Y | MR (Stepwise) | Age, Deprivation, General health, Physical function, Pain |  | Illness perceptions (inc. personal control), Coping, Personality, Distress | Distress β =0.32; IPQ Identity β = 0.44; Neuroticism β =0.34 |
| Steginga & Occhipinti 2006 | PC | Pre-Trt | 12 | Decisional distress (Decisional conflict scale) | Y | Hierarchical MR |  |  | Optimism, Cancer threat, Coping, Decisional distress | Decisional distress β =.515 |
| Lebel et al, 2008** | BC | Post-Trt | 72 | ED (POMS) | Y | Hierarchical MR (stepwise) | Age, Educ, 2nd cancer, Perceived health | Social support | Coping, Optimism, Fear of future, Stress appraisal , Emotional distress, Trauma | Coping (PPS) β =-0.28 |
| Barez et al 2009 | BC | Post-op | 12 | ED (HADS & POMS combined) | Y | LGC | Age , Trt, |  | Baseline Perceived control (PC) Change in PC | Change in perceived control β =-0.81; Intercept perceived control β =-0.31; Intercept distress β =-0.51; Intercept perceived control via change perceived control β =-0.28 |
| Risvedt & Trinkaus 2009 | RC | Post-op | 24-60 | EF (FACT) | N | LR | Gender, Age, Educ,TStage, Ostomy , Faecal incontinence |  | Trait anxiety | **EF :** Trait anxiety OR 1.45, |
| Scharloo et al 2010 | HN | Diag | 24 | EF (QLQ-C30 - EF) | Y | Hierarchical MR (Forced / stepwise) | Age, Tstage |  | Illness perceptions , EF | EF β =-0.65 |
| Carlson et al 2013^***^ | Mix | 1 month since diag | 12 | Improved Emotional distress  (Distress Thermometer) | Y | MR | Age, Gender, Source of income , Educ, Ethnic/cultural background  TSite , Trt | psychosocial resources, marstat, livstat, | Anx, Dep | **Distress DV:** Distress β =-0.56, No surgery β =0.08, |
| Hou & Lam, 2014 | RC | <12 weeks since diagnosis | 12 | ED  (HADS) | Y | Cross-Lagged Panel Model |  |  | ED  Well-being (WB:positive affect + life satisfaction) | EDT1- EDT2 b=.26 & EDT2-EDT3 b=.27;  WBT1-EDT2 b-.16 & EDT2-EDT3 b=.27;  WBT1-WBT2 b=.34 & WBT2 – EDT3 b=-.16 |
|  |  |  |  |  |  |  |  |  |  |  |
| Kohler et al, 2014 | PC | Pre-Trt | 12 post surgery | ED (HADS) | Y | MR | Concurrent urinary symptoms and erectile dysfunction |  | ED | ED = 0.48, Concurrent urinary symptomd β = 0.39 |
| Mehta et al 2003 | PC | Pre-Trt | NOT clear if prospective or cross-sectional | Fear of Cancer Recurrence (FCR) | N | MR | Age, Clinical characteristics, Trt, HRQoL,(SF-36) Symptoms |  |  | QoL-physical R^2^c = 0.27, QoL-mental R^2^c = 0.04 (no data) |

**N.B:** BC – Breast cancer, LC – Lung cancer, HN – Head & Neck cancer, PC – Prostate cancer, GC – Gynaecelogical cancer, RC – Rectal Cancer, Mix - heterogeneous cancer diagnoses; Diag – diagnosis; Op – Operation/surgery; Trt – Treatment; MD – Major Depressive Disorder; GAD – Generalised Anxiety Disorder; Anx – Anxiety; Dep - Depression; Trauma – Trauma symptoms; EF – Emotional Functioning; ED – Emotional distress; Opt – Optimism; ROC – Receiver Operating Curve; MR – Multiple regression, (M)LR- (Multinomial)Logistic regression; R^2^c - R^2^Change; β = Beta; OR = Odds ratio; HR = Hazards Ratio; R = Correlation; Only coefficients sig p<.05 are shown; HRQoL – Health related Quality of Life; TStage – Tumour stage; Tsize – Tumour size; TSite – Tumour site/clinical characteristics; Recur – cancer recurrence; Adj Trtr – adjuvant treatment; Chemo – Chemotherapy, Radio – radiotherapy, HT - Hormone Therapy; Educ – Education; Marstat – Marital status, Livstat – Living alone/with others; Empstat – Employment status; Grey font - cross-sectional
