# Supplementary material for: Predictors of emotional distress a year or more after diagnosis of cancer: A systematic review of the literature
Source: Psychooncology. 2018 Jan 10;27(3):791–801. doi: 10.1002/pon.4601 (PMC5873392; doi:10.1002/pon.4601)
Supplement: Supplementary file 3 — Table S3: Glossary of distress measures (DVs) used in included papers [file PON-27-791-s003.doc]

**Table 4: Glossary of distress measures (DVs) used in included papers**

| **Measure** | **Abbreviation** | **Outcome assessed** |
| --- | --- | --- |
| Present State Examination | PSE | Anxiety/ Depression Cases |
| Structured Clinical Interview | SCID | Major Depressive Disorder/ Generalized Anxiety Disorder |
| The Psychological Screen for Cancer | PSSCAN | Anxiety / Depression Cases |
| Hospital Anxiety and Depression Scale | HADS | Anxiety / Depression / Emotional Distress |
| The Center for Epidemiologic Studies Depression Scale | CES-D | Depression |
| Beck Depression Inventory | BDI | Depression |
| Hamilton Depression Rating Scale | HDRS | Depression |
| The Zung Self-Rating Depression Scale | Zung - SDS | Depression |
| Brief Symtom Inventory | BSI | Anxiety / Depression |
| Endler Multidimensional Anxiety Scales | EMAS-State | Anxiety |
| Post-traumatic Stress Disorder Checklist – Civilian version | PCL-C | Trauma Symptoms |
| Impact of Events Scale | IES | Trauma Symptoms |
| Harvard Trauma Questionnaire | HTQ | Trauma Symptoms |
| Stanford Acute Stress Reactions Questionnaire | SARSQ | Trauma Symptoms |
| Profile of Mood State | POMS | Emotional Distress |
| General Health Questionnaire | GHQ- 28 / GHQ 12 | Emotional Distress |
| Distress Thermometer | DT | Emotional Distress |
| The European Organization for Research and Treatment of Cancer (EORTC) Core Quality of Life Questionnaire – Emotional Functioning Scale | QLQ-C30 – EF | Emotional Functioning |
| Functional Assessment of Cancer Therapy-General – Emotional Functioning Scale | FACT-G - EF | Emotional Functioning |
| Affects Balance Scale | ABS | Emotional Distress |
| Decisional Conflict Scale | DCS | Decisional Distress |
| Fear of Recurrence Scale | FCR | Fear of Cancer Recurrence |
| Fear of Recurrence Scale | FOR | Fear of Cancer Recurrence |
